# Intra-Abdominal Sporadic Desmoid Tumors with Canonical *CTNNB1* and Uncommon Wnt/β-Catenin Pathway Somatic Mutations—A Clinicopathologic and Genetic Study of Five Cases

**DOI:** 10.3390/ijms27198655

**Published:** 2026-09-28

**Authors:** Chi Sing Ng, Jilong Qin, Gloria Y. Gao

**Affiliations:** 1 Department of Pathology, Caritas Medical Center, Wing Hong Street, Kowloon, Hong Kong; gyg550@ha.org.hk; 2 Department of Pathology, First Affiliated Hospital of Guangzhou Medical University, Guangzhou 510163, China; kyglkgyfyy@163.com

**Keywords:** desmoid tumor, *CTNNB1*, *APC*, *LRP6*, *TCF7L2*, Wnt/β-catenin pathway, in silico prediction

## Abstract

Desmoid tumors (DTs) may be sporadic or associated with familial polyposis syndromes, including familial adenomatous polyposis (FAP) and its variant, Gardner syndrome (GS). DTs are pathogenetically linked to activation of the Wnt/β-catenin signaling pathway. In sporadic DT, *CTNNB1* is most frequently implicated, with exon 3 somatic mutations (T41A, S45F and S45P) being the most commonly reported. *APC* is implicated in FAP/GS-associated DTs and occasionally in sporadic DT, most often through alterations involving the exon 15 mutation cluster region. In this study of five sporadic intra-abdominal DTs, we identified uncommon somatic missense mutations in three cases involving *APC* (exons 2 and 13), *LRP6* (exon 3) and *TCF7L2* (exons 3, 6, 8 and 10). One case harbored the canonical activating *CTNNB1* exon 3 p.S45P mutation, whereas another one case showed no detectable genomic alterations. Multi-algorithm in silico assessment using AlphaMissense, REVEL, CADD, SIFT and PolyPhen-2 provided supportive computational evidence for potential functional effects of several variants, particularly *TCF7L2* p.P113L, p.R243W and p.C337Y, and *CTNNB1* p.S45P, while predictions were variable for other variants. All five tumors arose intra-abdominally and presented as abdominal emergencies requiring surgical intervention. During a median follow-up of 24 months (range, 18–68 months), four patients remained recurrence-free, whereas one case developed multifocal intra-abdominal recurrence approximately five years after the initial resection and underwent repeat surgery. These findings broaden the spectrum of Wnt/β-catenin pathway alterations observed in intra-abdominal DTs, although the functional consequences of the uncommon variants require experimental validation.

## 1. Introduction

A DT is a non-metastasizing, locally aggressive neoplasm of fibroblasts and myofibroblasts. In sporadic DTs (SDTs), the most common somatic alterations are activating mutations in exon 3 of *CTNNB1* (approximately 89%) [1,2,3], although non-*CTNNB1* mutations affecting other genes and signaling pathways have also been documented [1,4,5,6]. In one study, 15.2% (31/204) of DTs harbored non-*CTNNB1* mutations involving genes such as *AKT*, *ALK*, *AR*, *EGFR*, *ERBB2*, *IDH2*, *KIT*, *RET*, *SDHA* and *SDHD*, several of which were predicted by in silico tools to be potentially deleterious [4]. Other studies have also reported BCL10, MPL and RBM10 alterations [1], as well as AKT, BRAF and TP53 mutations [5], sometimes co-occurring with *CTNNB1* alterations, rendering their contribution to DT pathogenesis difficult to interpret [1,4,5]. Dysregulation of Hedgehog, Notch, JAK/STAT, PI3K/AKT/mTOR, growth-regulatory and estrogen-related pathways has also been implicated in DTs [6]. DTs may occur in association with FAP and its variant GS, which are characterized by germline APC alterations [3,7]. Somatic APC mutations, however, have also been demonstrated in FAP-associated DTs [8,9] and sporadic DTs [9,10]. Furthermore, non-*CTNNB1* and non-*APC* somatic alterations have been reported in DT, including tumors with wild-type *CTNNB1* and *APC* [1,4,5,11,12,13]. In the present study, we identified uncommon somatic alterations in *APC* and the Wnt/β-catenin pathway genes *LRP6* and *TCF7L2* in three sporadic intra-abdominal DTs. The cohort is also notable for comprising exclusively intra-abdominal DTs, an uncommon anatomic subset [1], and for presentation as abdominal emergencies, which has been described only in a limited number of reports [14,15,16,17]. To strengthen the interpretation of the uncommon missense variants, we additionally performed a multi-algorithm in silico functional assessment.

## 2. Results

### 2.1. Clinicopathologic, Immunohistochemical and FISH Findings

There were three females and two males, none of whom had a personal or family history of intestinal polyposis or DT. One female patient (Case 2) had a history of pregnancy 18 years before the abdominal presentation. None of the three female patients had used oral contraceptives. None of the five patients had a prior history of abdominal surgery, radiotherapy or trauma before their initial DT presentation. All five patients presented with intractable abdominal pain and underwent surgery for large obstructive small-intestinal tumors arising from the mesentery and extensively involving the intestinal wall, in some cases reaching the mucosa. Tumor size ranged from 3.5 to 13 cm. Grossly, the tumors were firm and whitish with irregular borders. All resection margins were free of tumor. Histologically, the tumors comprised bland spindle cells arranged in fascicles and showed varying proportions of seven previously described histologic patterns [1] (Table 1 and Figure 1). Four cases exhibited all seven patterns. Cytologic atypia and mitotic activity were consistently absent. The growth pattern was uniformly infiltrative, with involvement of the mesentery and intestinal wall, in some areas extending almost transmurally and focally into the mucosa. Immunohistochemically, tumor cells in all five cases showed moderate nuclear β-catenin staining (Figure 1). All tumors were positive for α-SMA, and three showed desmin expression. STAT6 was negative in all tested cases. The remaining tested markers were negative. The clinicopathologic and immunohistochemical findings are summarized in Table 1 and Table 2. No USP6 rearrangement was detected by FISH in any of the five cases (Figure 1L).

#### Postoperative Follow-Up and Recurrence

All five patients underwent surgical resection with negative resection margins and were subsequently managed by observation without adjuvant therapy. The median follow-up duration was 24 months (range, 18–68 months). Four patients (Cases 1–4) remained free of recurrence during follow-up. Case 5 developed multifocal intra-abdominal recurrence approximately five years after the initial surgery. Follow-up imaging in September 2025 demonstrated newly developed soft-tissue lesions involving the operative bed, mesenteric region and left anterior abdominal wall. The patient underwent repeat surgical resection on 20 September 2025, with histopathologic confirmation of recurrent desmoid-type fibromatosis. At the most recent imaging assessment in April 2026 and clinical follow-up in May 2026, no further recurrence was documented. Detailed postoperative management and follow-up information is summarized in Table 3.

### 2.2. Genetic Variants

Case 1 showed no detectable genetic alteration. The genomic alterations identified in the remaining four cases are summarized in Table 4 Paired analysis of tumor and non-neoplastic intestinal tissue confirmed that the listed alterations were somatic: they were detected in tumor tissue but not in the matched non-neoplastic tissue. None of these alterations was identified as a corresponding population variant in gnomAD v4.1.1. All five tumors showed low tumor mutational burden (TMB) and were microsatellite-stable.

#### 2.2.1. *CTNNB1*

A *CTNNB1* mutation was detected in Case 3. This was the activating exon 3 hotspot mutation c.133T>C (p.S45P), a recognized canonical driver alteration in DT.

#### 2.2.2. *APC*

An *APC* exon 13 missense mutation and an exon 2 missense mutation were detected in Cases 5 and 2, respectively. These alterations are distinct from the more typical pathogenic *APC* alterations involving the exon 15 mutation cluster region (MCR).

#### 2.2.3. Other Wnt/β-Catenin Pathway Genes

##### *LRP6* 

A Tier 3 LRP6 missense (MS) mutation, c.619C>T (p.H207Y), was detected in Case 5. Its pathogenetic significance in DTs is uncertain.

##### *TCF7L2* 

Tier 3 *TCF7L2* MS mutations involving exons 3, 6, 8 and 10 were detected in Cases 3, 4 and 5. Their pathogenetic significance in DTs is uncertain.

#### 2.2.4. In Silico Functional Prediction

To further assess the potential functional relevance of the Wnt/β-catenin pathway MS variants identified by WGS, we integrated predictions from AlphaMissense, REVEL, CADD, SIFT and PolyPhen-2 (Table 5). *TCF7L2* p.P113L, p.R243W and p.C337Y showed strong concordant computational evidence for a deleterious effect across multiple algorithms. *TCF7L2* p.K283N was also predicted to be deleterious by AlphaMissense, REVEL, CADD and SIFT, although PolyPhen-2 classified it as benign. *CTNNB1* p.S45P showed predominantly deleterious predictions, including an AlphaMissense score of 0.9557 and CADD PHRED score of 29.2. *APC* p.E26K was predicted to have potential functional impact by several algorithms, whereas *APC* p.G600D was predominantly predicted to be benign/tolerated. *LRP6* p.H207Y showed mixed predictions, with REVEL, CADD, SIFT and PolyPhen-2 supporting a potential deleterious effect despite an ambiguous AlphaMissense classification. These predictions provide supportive computational evidence but do not constitute experimental demonstration of pathogenicity or pathway activation.

## 3. Discussion

DT/fibromatosis is a rare fibroblastic and myofibroblastic neoplasm with an estimated incidence of 4–6 cases per million persons per year, accounting for approximately 0.03% of all tumors and 3% of soft-tissue tumors [3]. Sporadic DT, which accounts for the majority of cases, most commonly harbors somatic activating *CTNNB1* mutations at 3p22.1 [1]. The most frequently reported hotspot alterations involve exon 3, particularly p.T41A, p.S45F and p.S45P [1,2,3,8,11,12,18,19]. In our cohort, paired analysis of tumor and matched non-neoplastic intestinal tissue confirmed the somatic nature of the reported alterations, which were also cross-referenced against gnomAD v4.1.1. Hereditary DTs are most often associated with FAP and germline inactivating *APC* alterations at 5q21-22, frequently involving the exon 15 MCR [7,8]. Somatic *APC* alterations have also been reported in FAP-associated DTs [8,9] and in sporadic DTs [9,10]. Canonical *CTNNB1* and pathogenic *APC* alterations converge on activation of the Wnt/β-catenin pathway and are generally mutually exclusive as primary drivers [1,3,13]. The additional Wnt-pathway variants identified in our cases should therefore be interpreted cautiously as potential modifiers or alternative pathway alterations until their functional consequences are experimentally established.

### 3.1. Mechanism of Nuclear β-Catenin Accumulation and Wnt Signaling

In DT, impaired β-catenin degradation results in nuclear β-catenin accumulation and downstream Wnt/β-catenin pathway activation. Canonical Wnt signaling is initiated by binding of Wnt ligands to Frizzled receptors together with the low-density lipoprotein receptor-related proteins LRP5/6. In the absence of Wnt signaling, the β-catenin destruction complex, composed principally of AXIN, APC, GSK3β and casein kinase 1, promotes sequential phosphorylation and ubiquitin-mediated proteasomal degradation of β-catenin [20,21,22,23,24]. Wnt pathway activation inhibits this degradation process, allowing β-catenin to accumulate and translocate to the nucleus. Activating *CTNNB1* mutations at critical phosphorylation sites reduce β-catenin degradation, whereas pathogenic APC alterations impair destruction-complex function [6,7,21,22,23]. Nuclear β-catenin interacts with TCF/LEF transcription factors to regulate downstream targets including *MYC*, *CCND1*, CD44 and *IGF1*, thereby influencing cell proliferation and other oncogenic programs [22,23,25,26].

### 3.2. Genetic Alterations

#### 3.2.1. *CTNNB1* Mutations

Among reported exon 3 CTNNB1 mutations in SDTs, p.T41A accounts for approximately 55–60%, p.S45F for 20–22% and p.S45P for 9–12% [1]. The p.S45F alteration has been associated with a higher risk of recurrence and poorer response to meloxicam in some studies [18,19,26]. The least common p.S45P mutation detected in Case 3 is a recognized activating hotspot and may be associated with less likelihood of tumor recurrence. This patient remained recurrence-free at the latest clinical follow-up 19 months after surgery. In silico analysis in the present study also supported a deleterious functional effect of p.S45P, although computational prediction is not required to establish its known canonical driver status.

#### 3.2.2. *APC* Mutations

*APC* MS mutations involving exons 2 and 13 were detected in two cases. These alterations differ from the pathogenic somatic or germline APC variants that most often involve exon 15, particularly the MCR [7,8,9,10]. Rare APC variants outside the MCR have nevertheless been described, including a germline alteration at the extreme 3′ end of exon 15 associated with a severe desmoid phenotype [27] and uncommon non-exon 15 alterations reported in abdominal DTs [28]. In our computational analysis, *APC* p.E26K showed predominantly deleterious predictions, whereas p.G600D was predicted to be predominantly benign/tolerated. Accordingly, the biological significance of these noncanonical *APC* MS variants remains uncertain and should not be inferred solely from their location within *APC* or from computational prediction.

#### 3.2.3. Other Wnt/β-Catenin Pathway Gene Mutations

##### *LRP6* 

Low-density lipoprotein receptor-related protein 6 (LRP6) is a single-pass transmembrane protein that, together with LRP5 and Frizzled receptors, functions as a Wnt co-receptor and contributes to canonical Wnt/β-catenin signaling [22,23,24,25,26,29]. LRP6 overexpression, hyperphosphorylation or pathway upregulation has been described in several carcinomas (breast, colorectal, esophageal, hepatocellular, pancreatic oral, urinary bladder and kidney), and *KRAS*-mutated cells in culture. This may enhance Wnt/β-catenin signaling as well as intersect with other signaling networks [30,31,32]. Single nucleotide polymorphism affecting *LRP6* has also been reported in several human diseases and cancers [33]. In our cohort, *LRP6* p.H207Y showed mixed in silico predictions: AlphaMissense classified the variant as ambiguous, whereas REVEL, CADD, SIFT and PolyPhen-2 supported a potential deleterious effect. These findings raise the possibility that p.H207Y may influence LRP6 function, but they do not establish that this variant caused β-catenin accumulation or acted as a driver in DT. Functional studies will be required to determine its actual effect on Wnt signaling.

##### *TCF7L2* 

*TCF7L2* belongs to the TCF/LEF family of high-mobility-group transcription factors, which also includes TCF7/TCF1, TCF7L1/TCF3 and LEF1 [34] These nuclear factors interact with β-catenin to regulate Wnt-responsive transcription [22,23,24,25,26]. *TCF7L2* has been implicated in several human cancers, including colorectal and gastric cancers, breast and lung carcinomas, gliomas, and hepatoblastomas [34,35,36]. Depending on cellular context, altered TCF7L2 activity may affect *MYC*, cell-cycle regulators, adhesion programs and invasive behavior [37,38,39,40,41,42]. The *TCF7L2* missense variants identified in our DT cases involved exons 3, 6, 8 and 10. Multi-algorithm in silico analysis provided strong concordant evidence for potential deleterious effects of p.P113L, p.R243W and p.C337Y; p.K283N was predicted to be deleterious by four of five algorithms but benign by PolyPhen-2. These data strengthen the rationale for considering *TCF7L2* as a potentially relevant Wnt-pathway alteration in this cohort, but they do not demonstrate that these variants are oncogenic drivers or directly activate Wnt/β-catenin signaling. This distinction is particularly important in Case 3, which also harbored the canonical *CTNNB1* p.S45P driver. Functional validation will be necessary to define the biological consequences of the *TCF7L2* variants in DT.

#### 3.2.4. Interpretation of In Silico Predictions

The use of several complementary algorithms was intended to reduce reliance on any single prediction framework. Concordant predictions across AlphaMissense, REVEL, CADD, SIFT and PolyPhen-2 provide supportive evidence that several uncommon variants may affect protein function; however, these tools were developed primarily to estimate variant deleteriousness rather than to prove tumor-specific pathway activation. Discordant predictions, as observed for *TCF7L2* p.K283N and *LRP6* p.H207Y, further emphasize this limitation. Accordingly, the computational results should be regarded as hypothesis-generating evidence that prioritizes variants for future functional testing rather than as proof of pathogenicity in DT.

#### 3.2.5. Possible Other CTNNB1 and APC Genetic Alterations

Notably, Case 1 showed nuclear β-catenin accumulation despite the absence of a detectable somatic mutation. This genotype–phenotype discrepancy may have several explanations. Previous studies have demonstrated that some desmoid tumors initially classified as wild type harbor low-frequency *CTNNB1* mutations or *APC* alterations that become detectable with more sensitive genomic approaches [12]. Moreover, intragenic deletions involving *CTNNB1*, which may be missed by conventional sequencing pipelines, have been identified in apparently wild-type desmoid tumors [43]. These observations indicate that the absence of a detectable mutation does not necessarily exclude an underlying genomic mechanism of Wnt/β-catenin activation.

#### 3.2.6. Epigenetic Mechanisms

Beyond cryptic genomic alterations, non-mutational regulation of Wnt/β-catenin signaling provides another biologically plausible explanation for nuclear β-catenin accumulation in an apparently mutation-negative tumor. Gene-expression profiling has demonstrated transcriptional heterogeneity between *CTNNB1*-mutant and wild-type desmoid tumors, indicating that distinct transcriptional programs accompany different *CTNNB1* genotypes [44]. Epigenetic heterogeneity has also been demonstrated in DTF by genome-wide DNA methylation profiling, although the available data do not establish DNA methylation as a driver of β-catenin activation in mutation-negative tumors [45]. More broadly, Wnt/β-catenin signaling is subject to multilayered epigenetic regulation through DNA methylation, chromatin and histone modifications, and non-coding RNAs [46]. A functional link between these processes is further supported by experimental evidence that DNA methyltransferase 1 (DNMT1) can regulate nuclear β-catenin abundance and β-catenin/TCF-dependent transcription, while perturbation of the DNMT1–β-catenin interaction can alter DNA methylation [47]. Differential TCF7L2 isoform balance and activity can modulate Wnt signaling [31], and differential interactions between β-catenin and TCF/LEF transcription factors may alter pathway output [33]. Promoter hypermethylation of Wnt antagonists and changes involving tumor-microenvironmental growth factors, inflammatory mediators and extracellular-matrix proteins have also been proposed as epigenetic contributors to DT biology [6,34]. Collectively, these studies support the biological plausibility that transcriptional or epigenetic dysregulation could modulate Wnt/β-catenin activity in the absence of an identifiable canonical mutation. They therefore support the biological plausibility of a non-mutational mechanism in Case 1, but do not establish such a mechanism in this particular tumor. In the absence of RNA-seqencing or methylation analysis, the basis of nuclear β-catenin accumulation in Case 1 remains hypothetical and unresolved.

Clinical observations also support a hormonal contribution to DT, including female predominance and an association with pregnancy in some patients [1,3,13]. Expression of estrogen receptor-β has been reported in DTs and provides a potential biologic basis for this association [6]. Prior surgery, irradiation or trauma have also been described as clinical associations [1], although their mechanistic relationship to DTs remains incompletely understood. In our series, Case 5 developed a recurrent DT five years after the initial resection.

### 3.3. Presentation, Diagnosis and Differential Diagnoses

Abdominal emergencies may result from intestinal obstruction, perforation, hemorrhage, acute peritonitis or tumor rupture and can require urgent surgical intervention. Intra-abdominal DTs are an uncommon cause of such presentations and often cannot be definitively diagnosed until histopathologic examination of resected tissue. Only a limited number of recent case reports have described intra-abdominal DTs presenting as an acute abdominal emergency [14,15,16,17], and most lacked comprehensive genomic characterization. The present series therefore adds molecularly characterized examples of this unusual clinical presentation.

DTs show a broad morphologic spectrum that must be recognized for accurate diagnosis. Seven histologic patterns have been described [1,35,48] conventional fascicular, hyalinized, staghorn vessel, hypocellular/myxoid, keloidal, nodular fasciitis-like and hypercellular. Individual tumors may contain several patterns. Our cases showed multiple patterns in varying combinations, and four displayed all seven. Across these patterns, the spindle fibroblastic/myofibroblastic cells were cytologically bland with inconspicuous nucleoli and minimal mitotic activity. The nodular fasciitis-like and staghorn vessel patterns were particularly important diagnostic pitfalls because they may suggest nodular fasciitis or solitary fibrous tumor, respectively.

Nuclear β-catenin expression is a useful diagnostic feature of DTs [1,19,49], although it is not entirely specific and may be observed in other mesenchymal tumors [50]. DTs may show nuclear β-catenin expression with or without a detectable *CTNNB1* or *APC* alteration [1,3,4,5,6,7,11,18,19,26,28]. Some studies have associated stronger nuclear β-catenin staining with adverse outcomes [19], but prognostic interpretation of staining intensity should be cautious. In our cases, nuclear staining was of moderate intensity. Accurate diagnosis therefore requires integration of clinical setting, morphology, immunophenotype and, when appropriate, molecular findings.

Nodular fasciitis is an important differential diagnosis, particularly for the nodular fasciitis-like areas of DT. It typically shows a tissue-culture-like growth pattern andxtravasated red blood cells, and lacks the characteristic nuclear β-catenin pattern of DT; recurrent *USP6* rearrangements provide a useful molecular discriminator [51]. *USP6* FISH was negative in all of our cases. Solitary fibrous tumor may also show keloidal collagen and staghorn vessels and can occasionally exhibit nuclear β-catenin staining, but it is characteristically nuclear STAT6-positive [52]. An inflammatory myofibroblastic tumor can be distinguished by its inflammatory background, variable cytologic features and the presence of ALK or other kinase rearrangements in a subset of cases [53]. Additional spindle-cell mimics include sarcomatoid carcinoma, melanoma, dedifferentiated liposarcoma, leiomyosarcoma, malignant peripheral nerve sheath tumor, synovial sarcoma, angiosarcoma, fibrosarcoma and gastrointestinal stromal tumor; the integration of morphology, immunophenotype and molecular findings facilitates their distinction from DTs [1].

### 3.4. Treatment and Prognosis

Active surveillance is an appropriate initial strategy for many asymptomatic or minimally symptomatic DT. However, intra-abdominal tumors can produce obstruction, bleeding or other complications that necessitate intervention. Reported recurrence risk varies with factors such as tumor size, age, anatomic site, β-catenin staining intensity and *CTNNB1* genotype [1,3,12,14,15,16,17,18,19,54,55,56,57,58]. Strong β-catenin staining intensity has been associated with worse prognosis [19], and the p.S45F genotype with a higher risk of recurrence in several studies [1,18,55,56,57]. The prognostic effect of resection-margin status is less consistent, although complete resection may reduce recurrence risk in selected surgically treated patients [1,12,28]. Because all patients in our series presented with abdominal emergencies, surgery was clinically indicated; all resections achieved negativeamargins. The tumor location may, however, be the most important prognostic factor, as indicated in one study [55]. During a median follow-up of 24 months (range, 18–68 months), four patients remained recurrence-free, whereas Case 5 developed multifocal recurrence approximately five years after initial surgery and was treated by repeat resection. Other systemic treatment options include non-steroidal anti-inflammatory drugs, anti-hormonal therapy, cytotoxic chemotherapy, tyrosine kinase inhibitors and γ-secretase inhibitors, ideally selected within a multidisciplinary framework [59,60,61]. Increasing understanding of canonical and noncanonical Wnt/β-catenin signaling and interacting pathways continues to inform the development of mechanism-based therapies [11,47].

### 3.5. Limitations of This Study

#### 3.5.1. Small Case Numbers

A major limitation of this study is the small number of cases involved. The five cases studied, however, represented a collection from three major centers over a 6-year period, reflecting the rarity of intra-abdominal DTs [1]. The small case number is further related to the uncommon presentation as abdominal emergencies [14,15,16,17]. Further work on more cases is warranted for better understanding of the genomics and epigenetics of DT.

#### 3.5.2. Functional and Epigenetic Validation

A further limitation is the absence of direct experimental validation of the uncommon Wnt/β-catenin pathway variants. Although multi-algorithm in silico predictions were performed, these analyses cannot establish variant-specific effects on β-catenin/TCF transcriptional activity and should be considered hypothesis-generating. Cell-based functional assays, transcriptome profiling and targeted DNA methylation studies were not performed. In cases such as our Case 1, the molecular basis of nuclear β-catenin accumulation remains unresolved because these analyses were not available to investigate potential non-mutational mechanisms of Wnt/β-catenin pathway activation. Future studies incorporating engineered functional models, RNA sequencing and methylation analyses in larger DT cohorts will be important to define the biological consequences of these uncommon alterations and to clarify possible epigenetic mechanisms.

## 4. Materials and Methods

### 4.1. Clinicopathologic and Immunohistochemical Study

Five SDTs presenting as abdominal emergencies were identified over a six-year period (2020–2025) from three major centers. Three cases were from the First Affiliated Hospital of Guangzhou Medical University, China, and one each from Caritas Medical Center and St. Teresa’s Hospital, Hong Kong. Clinical, imaging and follow-up records were reviewed. Tumor and matched non-neoplastic tissues were obtained from resection specimens and processed as formalin-fixed, paraffin-embedded (FFPE) blocks. Four-micrometer H&E-stained sections were prepared.

#### 4.1.1. Immunohistochemistry

Immunohistochemical staining was performed on formalin-fixed, paraffin-embedded tissue sections using fully automated immunostaining platforms, including the BOND-III Leica Biosystems, Mount Waverley, Victoria, Australia), Autostainer Link 48 (Dako/Agilent, Carpinteria, CA, USA), and BenchMark ULTRA (Ventana/Roche, Oro Vallry, AZ, USA), according to the manufacturers’ instructions and laboratory-validated protocols. Briefly, tissue sections were subjected to automated deparaffinization and heat-induced epitope retrieval, followed by incubation with the respective primary antibodies. Antigen–antibody complexes were visualized using the corresponding polymer-based detection systems with 3,3′-diaminobenzidine (DAB) as the chromogen, followed by hematoxylin counterstaining. Appropriate positive and negative controls were included in each staining run. Details of the primary antibodies, including clone, host species, working dilution, manufacturer, and the corresponding secondary antibody/detection system and automated immunostainer, are provided in Table 6.

#### 4.1.2. Postoperative Follow-Up and Assessment of Recurrence

Postoperative follow-up data were retrospectively obtained from the electronic medical records and radiologic imaging database. Follow-up included clinical assessment and cross-sectional imaging with computed tomography (CT) and/or magnetic resonance imaging (MRI), as clinically indicated. Postoperative recurrence was defined as the development of a newly detected or progressively enlarging soft-tissue lesion at or adjacent to the previous operative bed, or at an anatomically related intra-abdominal, mesenteric, or abdominal wall site, that was considered radiologically compatible with recurrent desmoid tumor on follow-up CT or MRI. Histopathologic confirmation was obtained when recurrent lesions were surgically resected. The date of recurrence was defined as the date of the first imaging examination demonstrating radiologic evidence of recurrent disease. For patients without recurrence, follow-up duration was calculated from the date of initial surgery to the date of the most recent available clinical or radiologic assessment. Detailed postoperative management and follow-up data are provided in Table 3.

### 4.2. Molecular Analyses

#### 4.2.1. Genomic DNA Preparation

Tumor tissue and matched non-neoplastic intestinal tissue from all five cases were analyzed. The non-neoplastic intestinal tissue served as the paired constitutional control for assessment of germline versus somatic status. Genomic DNA was extracted from FFPE tissue as previously described [62].

#### 4.2.2. Whole-Genome Sequencing (WGS)

Library construction and sequencing were performed as previously described [63]. Briefly, genomic DNA was fragmented using a Covaris E220 according to the manufacturer’s instructions. DNA fragments of approximately 100–300 bp were selected using AMPure XP beads (Agencourt, Bourgogne-Francher Comie, Fance), end-repaired, A-tailed and ligated to sequencing adapters. Adapter-ligated DNA was amplified by PCR and converted to a single-stranded circular DNA library. WGS was performed on the MGI Tech’s DNBSEQ-T7 platform, and sequencing-derived image files were processed by the MGI Tech’s DNBSEQ base-calling softwareto generate raw sequence data.

#### 4.2.3. Somatic Variant Annotation and Interpretation

SOAPnuke (version 2.2.6) was used for raw-data filtering. Reads were aligned to the UCSC human reference genome using the Burrows-Wheeler Aligner (BWA, version 0.7.17) and converted to binary alignment/map files. Variant analysis followed Genome Analysis Toolkit (GATK, version 4.4.0.0) best-practice workflows, including base-quality score recalibration, with quality-control procedures applied throughout the pipeline. Detected somatic variants were classified according to the joint consensus recommendations of the Association for Molecular Pathology, American Society of Clinical Oncology and College of American Pathologists [64]. gnomAD v4.1.1 was additionally consulted for population-frequency assessment.

#### 4.2.4. Fluorescence In Situ Hybridization (FISH) for *USP6*

*USP6* rearrangement testing was performed using a dual-color break-apart probe kit (Guangzhou Anbiping Pharmaceutical, Guangzhou, China), targeting sequences flanking chromosome 17p13.2. FFPE sections were deparaffinized, rehydrated, digested with pepsin, hybridized with the probes, and counterstained with 4′,6-diamidino-2-phenylindole. Signals were evaluated by fluorescence microscopy. Two hundred non-overlapping tumor nuclei were counted. Separation of the red and green signals by more than two signal diameters was considered a split signal, and cases with split signals in ≥15% of evaluable tumor nuclei were interpreted as positive for USP6 rearrangement.

#### 4.2.5. In Silico Functional Prediction

Missense variants involving *CTNNB1*, *APC*, *LRP6* and *TCF7L2* reported in this study were further assessed using the Ensembl Variant Effect Predictor (VEP) REST API with current VEP-supported RefSeq transcript versions and merged Ensembl/RefSeq annotation [65]. Five complementary prediction metrics were retrieved: AlphaMissense [66], REVEL [67], CADD PHRED [68], SIFT [69] and PolyPhen-2 [70]. AlphaMissense scores were interpreted using the published classification ranges: <0.34, likely benign; 0.34–0.564, ambiguous; and >0.564, likely pathogenic [66]. Predictions were interpreted collectively rather than on the basis of a single algorithm. These analyses were used to estimate potential functional impact and were not considered experimental evidence of pathogenicity or pathway activation (Table 5).

## 5. Conclusions

In conclusion, we report five molecularly characterized intra-abdominal DTs presenting as abdominal emergencies that required surgical intervention. In addition to a canonical activating *CTNNB1* exon 3 p.S45P mutation, uncommon somatic missense variants involving *APC*, *LRP6* and *TCF7L2* were identified. Multi-algorithm in silico analysis provided supportive computational evidence for potential functional effects of several variants, particularly *TCF7L2* p.P113L, p.R243W and p.C337Y, while other variants showed mixed or predominantly benign predictions. These data expand the observed spectrum of Wnt/β-catenin pathway alterations in DTs but do not establish the uncommon variants as drivers; experimental functional validation is required. During a median follow-up of 24 months (range, 18–68 months), four patients remained recurrence-free, whereas one patient developed multifocal recurrence approximately five years after the initial resection and underwent repeat surgical excision, with no further recurrence documented at the latest follow-up. Intra-abdominal DTs presenting as an abdominal emergency remain rare, and surgery may be unavoidable when obstruction, hemorrhage or other acute complications occur. Accurate diagnosis can be challenging because of the broad histologic spectrum and is best achieved through integrated clinical, morphologic, immunohistochemical and molecular assessment. Further mechanistic studies of Wnt/β-catenin pathway alterations may help refine biologic understanding and identify future therapeutic opportunities.

## Figures and Tables

**Figure 1:**
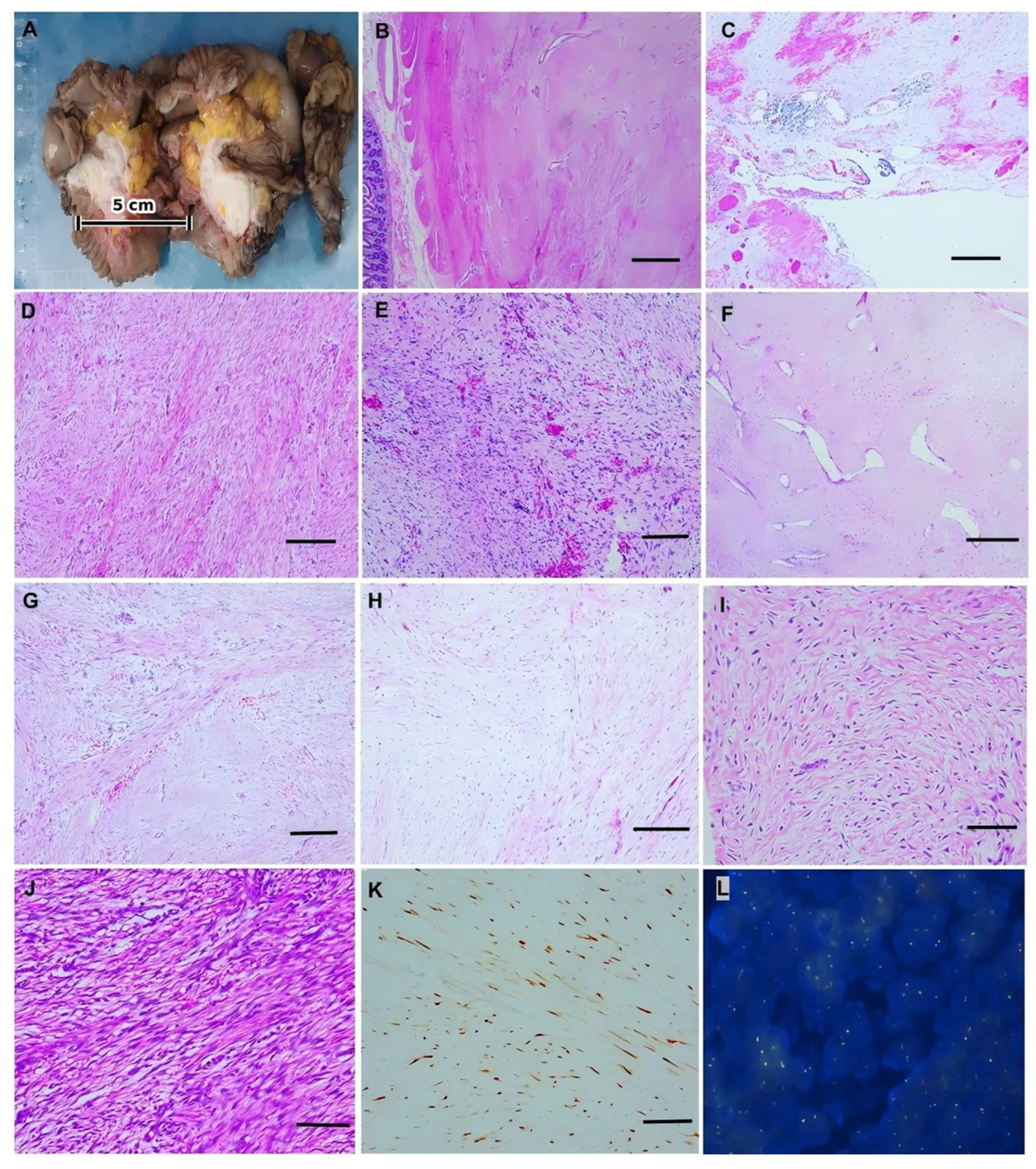
(**A**) Cut surface of mesenteric tumor of Case 5, featuring firm whitish tumor tissue extending to and involving the small-intestinal wall almost transmurally. (**B**) Low power revealed spindle cell tumor involving the muscularis propria and mesentery. H&E ×40, Scale bar: 500 μm. (**C**) Low power of mesenteric DTs involving adjacent jejunal wall with hemorrhage. H&E ×40, Scale bar: 500 μm. (**D**) Conventional pattern of DT. H&E ×200, Scale bar: 100 μm. (**E**) Nodular fasciitis-like pattern with extravasated red cells and loose stroma. H&E ×200, Scale bar: 100 μm. (**F**) Staghorn vessel pattern. H&E ×100, Scale bar: 200 μm. (**G**) Hyalinized pattern. H&E ×200, Scale bar: 100 μm. (**H**) Myxoid hypocellular pattern. H&E ×200, Scale bar: 100 μm. (**I**) Keloidal pattern, featuring thick ropy collagen fibers, H&E ×200, Scale bar: 100 μm. (**J**) Hypercellular pattern, with bland spindle cells in fascicles. H&E ×200, Scale bar: 100 μm. (**K**) Immunohistochemical staining for β-catenin showed tumor cell nuclear staining of moderate intensity. ×200, Scale bar: 100 μm. (**L**) *USP6* FISH showed no gene rearrangement.

**Table 1:** Clinicopathologic findings of 5 new sporadic intra-abdominal DT cases.

Case No.	Age	Sex	Clinical Presentation	Involved Sites	OC	* Previous Pregnancy	Previous Surgery or RT	Tumor Size	Resection Margins	Histology						
										C	Stag	N	Hyal	Myx	Kel	hyC
1	42	M	Abdominal pain, IO	Mesentery/Jejunum	NA	NA	No	13 cm	-ve	+	+	-	±	±	±	±
2	40	F	Abdominal pain, IO, weight loss	Mesentery/Jejunum	No	18 *	No	7 cm	-ve	+	+	+	±	±	±	±
3	57	F	Abdominal/back pain	Mesentery/Jejunum	No	No	No	4.5 cm	-ve	+	+	+	+	+	+	+
4	59	F	Abdominal pain, hematemesis	Mesentery/Jejunum	No	No	No	3.5 cm	-ve	+	-	+	+	±	+	+
5	32	M	Abdominal pain, melena	Mesentery/Duodenum/Abdominal wall	NA	NA	Y	13 cm	-ve	+	-	+	+	±	+	±

Abbreviations: C, conventional; hyal, hyalinized; hyC, hypercellular; IO, intestinal obstruction; kel, keloidal; myx, myxoid; N, nodular fasciitis-like; NA, not applicable; OC, oral contraceptive; RT, radiotherapy; Stag, staghorn vessel; Y, previous surgery 5 years ago before recurrence; +, >10%; ±, <10%; -, 0%; -ve, not involved; *: years before intra-abdominal disease.

**Table 2:** Immunohistochemical findings in 5 new sporadic intra-abdominal DT.

Case No.	β-Catenin	CK	α-SMA	Desmin	STAT6	PDGFRA	CD117	Dog1	S-100	SOX10	SS18-SSX	ALK(D5F3)	CD34
1	+	−	+/−	−	−	ND	−	−	−	ND	ND	ND	−
2	+	−	+/−	−	−	ND	−	−	−	ND	ND	ND	−
3	+	−	+	+/−	−	−	−	−	−	−	−	−	−
4	+	−	+	+/−	−	−	−	−	−	−	−	−	−
5	+	−	+	+/−	−	−	−	−	−	−	−	−	−

Abbreviations: α-SMA, α-smooth muscle actin; ND, not done; PDGFRA, platelet-derived growth factor receptor alpha; +, moderate positive; −, negative; +/−, weak or focal positive.

**Table 3:** Postoperative management, radiologic follow and recurrence status of the five patients with intra-abdominal desmoid tumors.

Case	Initial Surgery Date	Surgical Margin	Postoperative Management	Imaging Modality for Recurrence Assessment	Most Recent Imaging Date	Last Clinical Follow-Up Date	Follow-Up Duration (Months)	Recurrence Status	Date of Recurrence	Site/Pattern of Recurrence	Treatment for Recurrence	Status at Last Follow-Up
1	7 May 2024	Negative	Observation without adjuvant therapy	MRI	13 July 2025	November 2025	18	No	—	—	—	No evidence of recurrence
2	12 November 2023	Negative	Observation without adjuvant therapy	MRI	10 October 2025	November 2025	24	No	—	—	—	No evidence of recurrence
3	7 April 2024	Negative	Observation without adjuvant therapy	CT and MRI	9 August 2025	November 2025	19	No	—	—	—	No evidence of recurrence
4	30 August 2021	Negative	Observation without adjuvant therapy	CT	8 April 2025	November 2025	51	No	—	—	—	No evidence of recurrence
5	7 September 2020	Negative	Observation without adjuvant therapy	CT and MRI	2 April 2026	May 2026	68	Yes	September 2025	Multifocal recurrent disease involving the operative bed, mesenteric region, and left anterior abdominal wall	Repeat surgical resection, 20 September 2025	No evidence of further recurrence after repeat resection

Abbreviations: CT, computed tomography; MRI, magnetic resonance imaging. Follow-up duration was calculated from the date of initial surgery to the date of the most recent available clinical or radiologic assessment. When only the month and year of the last clinical follow-up were available, follow-up duration was calculated by calendar month.

**Table 4:** Genetic alterations in intra-abdominal DT cases.

Gene	Location	Alteration	Type	Tier	Case	VAF (%)	SIG
*CTNNB1*	exon3	c.133T>C:p.S45P	MS	1/2	3	15	S/P
*APC*	exon2	c.76G>A:p.E26K	MS	3	2	11	US
	exon13	c.1799G>A:p.G600D	MS	2/3	5	6	US
*LRP6*	exon3	c.619C>T:p.H207Y	MS	3	5	10	US
*TCF7L2*	exon3	c.338C>T:p.P113L	MS	3	5	29	US
	exon6	c.727C>T:p.R243W	MS	3	4	7	US
	exon8	c.849G>T:p.K283N	MS	3	3	20	US
	exon10	c.1010G>A:p.C337Y	MS	3	3	15	US

Abbreviations: MS, missense mutation; SIG, significance; S/P, strong/potential significance; US, unknown significance; VAF, variant allele frequency.

**Table 5:** In silico functional prediction of Wnt/β-catenin pathway missense variants identified by whole-genome sequencing.

Gene	RefSeq Transcript	cDNA Change	Protein Change	Case	VAF (%)	AlphaMissense Score/Class	REVEL	CADD PHRED	SIFT Score/Prediction	PolyPhen-2 Score/Prediction	Overall Interpretation
*CTNNB1*	NM_001098209.2	c.133T>C	p.S45P	3	15	0.9557/likely pathogenic	0.338	29.2	0.000/deleterious (low confidence)	0.909/probably damaging	Predominantly deleterious
*APC*	NM_000038.6	c.76G>A	p.E26K	2	11	0.9807/likely pathogenic	0.436	31.0	0.000/deleterious (low confidence)	0.993/probably damaging	Predominantly deleterious
*APC*	NM_001127511.3	c.1799G>A	p.G600D	5	6	0.2813/likely benign	0.298	23.0	0.080/tolerated (low confidence)	0.014/benign	Predominantly benign/tolerated
*LRP6*	NM_002336.3	c.619C>T	p.H207Y	5	10	0.4613/ambiguous	0.789	26.3	0.010/deleterious (low confidence)	0.945/probably damaging	Mixed; overall evidence favors potential deleterious effect
*TCF7L2*	NM_001349870.2	c.338C>T	p.P113L	5	29	0.8489/likely pathogenic	0.900	34.0	0.000/deleterious (low confidence)	0.993/probably damaging	Strong concordant evidence for deleterious effect
*TCF7L2*	NM_001349870.2	c.727C>T	p.R243W	4	7	0.9989/likely pathogenic	0.816	33.0	0.000/deleterious (low confidence)	0.995/probably damaging	Strong concordant evidence for deleterious effect
*TCF7L2*	NM_001349870.2	c.849G>T	p.K283N	3	20	0.9957/likely pathogenic	0.613	29.8	0.000/deleterious (low confidence)	0.203/benign	Predominantly deleterious; PolyPhen-2 discordant
*TCF7L2*	NM_001349870.2	c.1010G>A	p.C337Y	3	15	0.9998/likely pathogenic	0.816	33.0	0.000/deleterious (low confidence)	0.857/possibly damaging	Strong evidence for deleterious effect

Abbreviations: CADD, Combined Annotation-Dependent Depletion; REVEL, Rare Exome Variant Ensemble Learner; SIFT, Sorting Intolerant From Tolerant; VAF, variant allele frequency. Predictions were obtained through the Ensembl Variant Effect Predictor using current VEP-supported RefSeq transcript versions. AlphaMissense classes were interpreted as likely benign (<0.34), ambiguous (0.34–0.564), and likely pathogenic (>0.564). The overall interpretation summarizes concordance across multiple algorithms and should be regarded as computational evidence of potential functional impact rather than experimental confirmation of pathogenicity or Wnt/β-catenin pathway activation. Low-confidence SIFT labels are reported exactly as returned by VEP.

**Table 6:** Characteristics of antibodies and automatic immunostainers used for immunohistochemistry in the present study.

Antibody	Lab	Clone	Host Species	Dilution	Positive Cellular Localization	Manufacturer	Secondary Antibody/Detection System and Automated Immunostainer	Automated Immunostainer
CD117	FAH	YR145	Rabbit	RTU	Membranous/cytoplasmic	Maixin-BIO, Fuzhou, China	BOND Polymer Refine Detection (Leica Biosystems)	BOND-III
	C/S	Polyclonal	Rabbit	1:300		Dako, Glostrup, Denmark	Cocktail	ULTRA
β-catenin	FAH	MX043	Mouse	RTU	Nuclear	Maixin-BIO, Fuzhou, China	EnVision FLEX+ (Dako/Agilent)	Autostainer Link 48
	C/S	Β-catenin 1	Mouse	1:300		Dako, Glostrup, Denmark	Cocktail	ULTRA
S100	FAH & C/S	polyclonal	Rabbit	RTU	Cytoplasmic/nuclear	Dako, Glostrup, Denmark	EnVision FLEX+ (Dako/Agilent) Cocktail	Autostainer Link 48 & ULTRA
desmin	FAH C/S	D33 DE-R-11	Mouse Mouse	RTU RTU	Cytoplasmic	Dako, Glostrup, Denmark	EnVision FLEX+ (Dako/Agilent) Cocktail	Autostainer Link 48 ULTRA
α-SMA	FAH C/S	1A4 EP188	Mouse Rabbit	RTU 1:200	Cytoplasmic	Dako, Glostrup, Denmark Epitomics, Burlingame, CA, USA	EnVision FLEX+ (Dako/Agilent) Cocktail	Autostainer Link 48 ULTRA
SOX10	FAH	EP268	Rabbit	RTU	Nuclear	ZSGB-BIO, Beijing, China	EnVision FLEX+ (Dako/Agilent)	Autostainer Link 48
DOG1	FAH C/S	OTI1C6 K9	Mouse Mouse	1:200 1:40	Membranous/cytoplasmic	ZSGB-BIO, Beijing, China Leica, San Francisco, CA, USA	EnVision FLEX+ (Dako/Agilent) Cocktail	Autostainer Link 48 ULTRA
CD34	FAH C/S	10C9 OBEnd/10	Mouse Mouse	1:200 1:60	Membranous/cytoplasmic	ZSGB-BIO, Beijing, China Leica, San Francisco, CA, USA	BOND Polymer Refine Detection (Leica Biosystems) Cocktail	BOND-III ULTRA
STAT6	FAH C/S	EP325 YE361	Rabbit Rabbit	RTU 1:600	Nuclear	ZSGB-BIO, Beijing, China Abcam, Waltham, MA, USA	EnVision FLEX+ (Dako/Agilent) Cocktail	Autostainer Link 48 ULTRA
ALK	FAH	D5F3	Rabbit	RTU	Cytoplasmic	Ventana (Roche), Oro Valley, AZ, USA	OptiView DAB IHC Detection Kit (Ventana/Roche)	ULTRA
SS18-SSX	FAH	E9X9V	Rabbit	RTU	Nuclear	Maixin-BIO, Fuzhou, China	EnVision FLEX+ (Dako/Agilent)	Autostainer Link 48
CK	FAH C/S	CAM5.2 AE1/AE3	Mouse Mouse	RTU 1:200	Cytoplasmic	Maixin-BIO, Fuzhou, China Dako, Glostrup, Denmark	EnVision FLEX+ (Dako/Agilent) Cocktail	Autostainer Link 48 ULTRA
PDGFRA		polyclonal	Rabbit	RTU	Membranous/cytoplasmic	ZSGB-BIO, Beijing, China	BOND Polymer Refine Detection (Leica Biosystems)	BOND-III

Abbreviations: Cocktail, cocktail of horseradish peroxidase-labeled goat anti-mouse and goat anti-rabbit immunoglobulins (Ventanna ready-to -use second antibody); C/S, Caritas Medical Center/St. Teresa’s Hospital; Lab, laboratory; RTU, ready-to-use; ULTRA, Ventanna Benchmark Ultra.

## Data Availability

The original contributions presented in this study are included in the article. Further inquiries can be directed to the corresponding author.
